# Creatine in T Cell Antitumor Immunity and Cancer Immunotherapy

**DOI:** 10.3390/nu13051633

**Published:** 2021-05-13

**Authors:** Bo Li, Lili Yang

**Affiliations:** 1 Department of Microbiology, Immunology & Molecular Genetics, University of California, Los Angeles, Los Angeles, CA 90095, USA; 2 Eli and Edythe Broad Center of Regenerative Medicine and Stem Cell Research, University of California, Los Angeles, Los Angeles, CA 90095, USA; 3 Jonsson Comprehensive Cancer Center, The David Geffen School of Medicine, University of California, Los Angeles, Los Angeles, CA 90095, USA; 4 Molecular Biology Institute, University of California, Los Angeles, Los Angeles, CA 90095, USA

**Keywords:** creatine, T cell antitumor immunity, metabolic regulator, cancer immunotherapy

## Abstract

Creatine is a broadly used dietary supplement that has been extensively studied for its benefit on the musculoskeletal system. Yet, there is limited knowledge regarding the metabolic regulation of creatine in cells beyond the muscle. New insights concerning various regulatory functions for creatine in other physiological systems are developing. Here, we highlight the latest advances in understanding creatine regulation of T cell antitumor immunity, a topic that has previously gained little attention in the creatine research field. Creatine has been identified as an important metabolic regulator conserving bioenergy to power CD8 T cell antitumor reactivity in a tumor microenvironment; creatine supplementation has been shown to enhance antitumor T cell immunity in multiple preclinical mouse tumor models and, importantly, to synergize with other cancer immunotherapy modalities, such as the PD-1/PD-L1 blockade therapy, to improve antitumor efficacy. The potential application of creatine supplementation for cancer immunotherapy and the relevant considerations are discussed.

## 1. Introduction

Creatine (Cr) is a nitrogenous organic acid naturally occurring in vertebrates. Endogenous creatine is synthesized from arginine and glycine mainly in the kidney and liver by two enzymes, L-arginine:glycine amidinotransferase (AGAT; also known as GATM) and guanidinoacetate *N*-methyltransferase (GAMT). Synthesized creatine is released into the circulation and specifically taken up by the creatine transporter (CrT or SLC6A8) expressing cells, where creatine is further phosphorylated by creatine kinase (CK) using adenosine triphosphate (ATP) to form phosphocreatine (PCr) [1]. Four CK isoforms have been identified: two cytosolic isoforms, the muscle-type (CKM) and the brain-type (CKB); and two mitochondrial isoforms, the ubiquitous-type (CKMT1) and the sarcomeric-type (CKMT2) [2]. The CK/PCr/Cr system is coupled with ATP-adenosine diphosphate (ADP) transition to buffer intracellular ATP levels. PCr can be break down by CK to resynthesize ATP that supplies cellular energy needs in an acute manner [1].

Besides de novo synthesis, diet is also a primary source of natural creatine for humans. A person who eats red meat, poultry, and/or fish obtains approximately 50% of daily creatine need (about 2 g per day) from food intake [3]. Compared to non-vegetarians, vegetarians have significantly lower levels of creatine and phosphocreatine in the muscle [4,5]. The average creatine stores for a 70 kg adult are between 120 and 140 g [6]. However, approximately 2 g per day of creatine in the muscle are degraded into creatinine that is excreted in the urine [6]. Degradation of creatine is even greater in individuals with higher physical activity and/or larger muscle mass. Wallimann et al. have reported that normal omnivore diets generally provide 0.75–1.5 g/day of creatine and are not sufficient to fully saturate creatine stores in the body [7]. Therefore, creatine supplementation is an effective way to increase the creatine reservoir. Creatine is predominantly stored in skeletal muscle as free creatine (~40%) or phosphocreatine (~60%); the latter is a major source of bioenergy to the body [1].

Creatine was discovered as a constituent of meat by the French chemist Michel-Eugene Chevreul more than 180 years ago. However, creatine did not gain wide attention until the 1990s when two gold medalists from the 1992 Barcelona Olympics credited it with helping them enhance performance. The phosphagen energy metabolic system produces ATP more rapidly than other metabolic systems, such as the glycolysis system and the aerobic system [8]. Concentrations of cellular PCr in some tissues such as muscles or the brain can reach up to 30–40 mM, resulting in rapidly replenishing ATP stores that can be immediately used during high-energy demand states [9]. Over the past three decades, oral creatine supplements have been broadly utilized by athletes to improve performance during high-intensity exercise with repeated bouts and short rest periods, such as running, swimming, sprinting, jumping, and strength training [9,10,11,12,13]. The improvement in performance can reach up to 20% on various high-intensity exercise tasks [10]. A major mechanism is that increasing the stores of PCr in skeletal muscle leads to the rephosphorylation of ADP to ATP during bursts of high intensity exercise, thereby increasing the availability of bioenergy [14,15]. Hydrogen ions resulting from increased lactic acid accumulation during high-intensity exercise is a key contributor to muscle fatigue. Given that the breakdown of PCr to creatine and phosphate consumes a hydrogen ion, creatine can also buffer the pH changes caused by increased hydrogen ion concentration and maximize performance [16].

In addition to its use by athletes, creatine is the most popular nutrition supplement used by body builders to gain muscle mass [14]. The popular form of creatine used by athletes and body builders is creatine monohydrate [17]. The dosing strategy typically consists of two phases: the first phase is a loading phase in which athletes ingest 20 g per day of creatine over four doses for five to seven days, followed by a maintenance phase where 1–10 g per day of creatine is administered for a month or much longer [1,10]. The loading phase has been reported to increase muscle stores of PCr between 20% and 40% [9]. Importantly, the creatine-loading phase results in limited side effects, such as cramping, nausea, and fluid retention [18]. Alternatively, a line of evidence has indicated that the ingestion of 3 g per day of creatine for a minimum 28 days is as effective as the higher dose-loading regimen at raising total skeletal muscle creatine stores [19]. Gradually increasing the muscle stores of creatine may alleviate side effects that are commonly associated with the higher dose-loading regimen.

Despite 95% of creatine stores in skeletal muscle, a significant amount of creatine exists in the brain [20], where creatine plays key roles in maintaining normal neurological functions. Human genetic deficiencies in creatine pathway components involved in creatine synthesis or transport result in decreased levels of ATP in the brain, which is associated with various clinical symptoms including developmental delays, speech impairment, and mental retardation [21]. Several lines of evidence have revealed that oral creatine supplements protect against neurological disorders such as traumatic brain injury [22], amyotrophic lateral sclerosis [23], Huntington’s Disease [24], and Parkinson’s Disease [25].

Clinically, patients with deficiency in creatine synthesis can benefit from creatine supplementation; however, no treatment is available to patients with deficiency in the creatine transporter gene *CrT* [26,27]. The gene *CrT* is located on the X chromosome. This gene encodes the solute carrier family 6 member 8 (SLC6A8) that is a plasma membrane protein whose function is to transport creatine into and out of cells in a sodium- and chloride-dependent manner. A high expression of *CrT* gene is required for normal physiological functions of high-energy demanding cells and organs such as muscle and the brain [1]. Patients harboring human *CrT* mutations have been associated with a group of muscle and brain disorders [1]. In line with the clinical data, mice with systemic CrT deficiency have smaller body weight and exhibit deficits in memory and spatial learning [28]. However, the function and regulation of CrT/creatine system outside of the muscle and the brain remain largely elusive. New concepts of creatine biology in other systems and cell types have just begun to be developed in the past few years.

In this review, we aim to summarize recent advances in understanding creatine regulation of antitumor immunity, a topic that has previously gained little attention in the creatine research field. We also discuss the potential application of creatine supplementation for cancer immunotherapy and relevant considerations.

## 2. Creatine Regulation of T Cell Antitumor Immunity

T cells play a central role in mediating immune responses against cancer. In a tumor, CD8 cytotoxic T cells are activated through their T cell receptor (TCR) recognition of tumor antigenic peptides presented by major histocompatibility complex (MHC) class I molecules on tumor cells. Activated CD8 T cells can efficiently kill tumor cells by releasing cytotoxic molecules (e.g., granzymes and perforin) or using death ligands (e.g., TNF-related apoptosis-inducing ligand and CD95 ligand) [29,30]. Therefore, CD8 T cells have emerged as attractive therapeutic targets for cancer treatment [31,32,33,34].

The activation and proliferation of T cells are energy-demanding activities, which require large amounts of energy in the form of ATP [35]. Distinct metabolic programs such as glycolysis and tricarboxylic acid (TCA) cycle are utilized to convert nutrients (e.g., glucose, amino acids, and lipids) into ATP to support CD8 T cell homeostasis and effector function [35,36,37]. However, in the tumor microenvironment, T cells face fierce competition with fast-growing tumor cells for the limited availability of nutrients [38]. Therefore, an economical and efficient energy metabolism is required for tumor-infiltrating T cells to sustain effective antitumor responses [39]. However, metabolic regulators of T cell antitumor immunity remains poorly understood [40,41,42,43]. Studies of the metabolic regulation of T cell antitumor immunity thus may identify new therapeutic targets for treating cancer.

By using a B16-OVA mouse melanoma model, Di Biase and colleagues observed an upregulation of *CrT* gene expression in tumor-infiltrating CD8 T cells compared to CD8 T cells isolated from tumor-free mice. Further study of the tumor-infiltrating CD8 T cell subsets revealed an upregulation of *CrT* gene expression that was much more significant in the PD-1^hi^ cells compared to the PD-1^lo^ cells; the highest levels of *CrT* gene expression were detected in the PD-1^hi^Tim-3^hi^LAG-3^hi^ subset that is considered to be the most “exhausted” [44]. These findings suggest a possible feedback loop in tumor-fighting CD8 T cells that compensates for bioenergy-insufficiency by increasing creatine uptake; in particular, the “exhausted” CD8 T cells are highly capable of uptaking creatine and may benefit the most from creatine supplementation treatment [44].

To study if the CrT/creatine system regulates the energy metabolism of tumor-fighting CD8 cytotoxic T cells, systemic *CrT*-knockout (KO) mice were used for the B16-OVA tumor challenge. Notably, *CrT*-KO mice with creatine supplementation exhibited accelerated tumor growth compared to their *CrT*-wild-type (WT) littermates with creatine supplementation [44]. In line with these results, *CrT*-KO tumor-infiltrating CD8 T cells expressed higher levels of PD-1 compared to their wildtype counterparts [44], suggesting CrT deficiency may lead to bioenergy insufficiency and exhaustion of antitumor T cells.

To study if creatine directly or indirectly regulates antitumor immunity, WT CD45.1 congenic mice were reconstituted with bone marrow cells from either *CrT*-WT or *CrT*-KO donor mice and then challenged with B16-OVA melanoma cells. Indeed, CrT deficiency impeded the capacity of the reconstituted immune system to control tumor growth. Furthermore, OT1 transgenic (Tg)/*CrT*-KO mice were generated to produce OVA-specific CD8 T cells deficient in CrT. After transferring these T cells into WT CD45.1 congenic mice bearing pre-established B16-OVA tumors, they were less effective in suppressing tumor growth compared to control WT T cells [44]. Similarly, the OT1 Tg/*CrT*-KO CD8 T cells upregulated PD-1 levels and produced a smaller amount of effector T cell cytokines (e.g., IL-2 and IFN-γ) [44]. After in vitro antigen stimulation, *CrT*-KO CD8 T cells exhibited a dramatic reduction in cell proliferation, surface activation marker production (e.g., CD25), and effector cytokine (e.g., IL-2 and IFN-γ) and cytotoxic molecule (e.g., Granzyme B) production, whereas overexpression of *CrT* gene rescued the proliferation and function of *CrT*-KO CD8 T cells. Study of tumor antigen-specific CD8 T cells deficient in CrT exhibited similar in vitro results [44].

Taken together, these studies strongly support a new role of the creatine/CrT pathway in positively regulating an effector CD8 T cell response against tumor.

## 3. Molecular Mechanisms Mediating Creatine Regulation of CD8 T Cell Responses

Muscle cells and brain cells power cellular activities by using creatine to buffer intracellular ATP levels via the CK/PCr/Cr system [1]. Recent evidence has indicated that the expression levels of the *CrT* gene and *Ckb* gene are markedly upregulated in CD8 T cells after TCR stimulation [44], suggesting activated CD8 T cells may use a similar mechanism to power T cell activities, in particular antitumor reactivity. Compared to *CrT*-WT CD8 T cells, *CrT*-KO CD8 T cells contained significantly reduced levels of ATP after antigen stimulation. Supplementing ATP to T cell culture not only rescued hypoactivation of *CrT*-KO CD8 T cells, but also further activated *CrT*-WT CD8 T cells [44]. Another research group also reported that overexpression of the *Ckb* gene enhanced the cytokine production and proliferation of mature T cells, while blockade of CKB using specific CK inhibitor or Ckb short hairpin RNA (shRNA) resulted in severe impairment of T cell function [45]. Collectively, these results indicate that the CK/PCr/Cr ATP-buffering system is essential for a productive CD8 T cell response to antigen stimulation.

Besides the kidney and liver cells that are responsible for the classical pathway of creatine synthesis, other cell types, such as the muscle cells [46], adipocytes [47], and pancreatic acinar cells [48], have been indicated to synthesize creatine. Could *de novo* synthesis be another source to feed the CK/PCr/Cr system in T cells? To address this, expression of two genes encoding the key enzymes controlling creatine synthesis, *Agat* and *Gamt,* was examined in T cells. Both genes were expressed at low levels in CD8 T cells, and the expression of *Gamt* gene was further downregulated after TCR stimulation. Intracellular creatine levels were undetectable in activated *CrT*-KO CD8 T cells [44]. Hence, activated CD8 T cells may have a very limited capacity for *de novo* creatine synthesis and mainly rely on uptaking extracellular creatine through CrT to sustain the CK/PCr/Cr ATP-buffering system.

TCR stimulation leads to a cascade of phosphorylation events in a sequential manner in T cells [49]. Given that ATP supplies the phosphate group for the phosphorylation reactions catalyzed by the protein kinases [40], the intracellular ATP levels may have a strong impact on T cell activation signaling pathways. Creatine uptake deficiency in CD8 T cells dampened activation of the TCR proximal signaling molecule zeta chain of TCR-associated protein kinase 70 (Zap-70) and the downstream transcription factors, NFAT and AP-1. Supplementing ATP to T cell culture rescued the impaired TCR signaling in *CrT*-KO CD8 T cells [44]. Creatine supplementation further enhanced the phosphorylation of Zap-70 in *CrT*-WT CD8 T cells. Interestingly, the activation of another key downstream transcription factor, NF-κB, was not effected by creatine uptake deficiency [44], suggesting that the NF-κB signaling pathway may better resist ATP fluctuation during T cell response. Together, these data suggest that a CD8 T cell requires the CK/PCr/Cr ATP buffering system to properly activate TCR signaling pathways in response to antigen stimulation.

5′ Adenosine monophosphate-activated protein kinase (AMPK) can function as a nutrient and energy sensor to maintain cell energy homeostasis by detecting shifts in the AMP:ATP ratio. AMPK is activated in cells with low energy status. In order to restore cell energy homeostasis, activated AMPK stimulates ATP-producing catabolic pathways and inhibits ATP-consuming anabolic pathways. While AMPK as a key metabolic regulator plays critical role in T cell metabolism and function [50,51,52,53], excessive AMPK activation impairs the function of antigen-specific CD8 T cells [54]. In *CrT*-KO CD8 T cells, the decreased ATP levels were associated with increased activation of AMPK. In addition, AICAR, an AMPK activator, significantly suppressed activation of AP-1 transcription factor as well as production of effector T cell cytokines and surface activation markers in *CrT*-WT CD8 T cells [44]. Therefore, creatine-mediated ATP buffering system may enhance effector CD8 T cell activities in part through AMPK regulation of TCR signaling pathways. Further studies are necessary to understand whether creatine-mediated ATP/energy buffering system cross-regulates other signaling pathways and transcriptional events to augment antitumor T cell function.

## 4. Creatine Supplementation for Cancer Therapy—Potential Application for Cancer Immunotherapy

**Figure 1:**
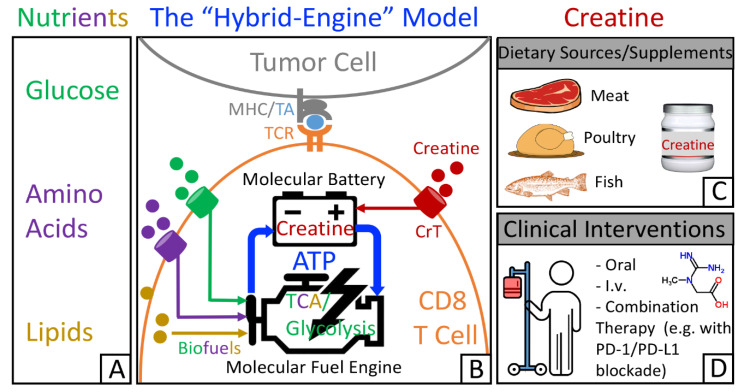
The “hybrid-engine” model in T cell antitumor immunity and potential application of creatine supplementation for cancer immunotherapy. (**A**) Limited nutrients for immune cells in the tumor microenvironment. (**B**) The “hybrid-engine” model. Analogous to the hybrid car, a tumor antigen-specific CD8 T cell uses a “molecular fuel engine”, including tricarboxylic acid cycle and glycolysis, to convert nutrients into adenosine triphosphate (ATP), while utilizing creatine-mediated energy buffering system, a “molecular battery”, to store ATP and power T cell activities. (**C**) Creatine can be obtained from creatine-rich diet (e.g., red meat, poultry, and fish) and dietary supplements. (**D**) Creatine administration as a therapeutic intervention would result in the best therapeutic effects. ©2019 Di Biase et al. Originally published in *J. Exp. Med.* doi: 10.1084/jem.20182044.

Many cancer immunotherapies have been designed to target immune cell metabolism in the tumor microenvironment [38,40,41,42,55]. For example, immune checkpoint blockade (ICB) therapies, such as PD-1/PD-L1 therapies, reduce tumor glycolysis and switch the energy metabolism to favor T cells [32,43,56,57,58]. By linking creatine to antitumor T cell activities, recent findings update the picture of the metabolic regulatory network that controls T cell antitumor immunity. A “hybrid-engine” model has been proposed (Figure 1A,B), in which a tumor antigen-specific CD8 T cell uses glucose, amino acids, and lipids as fuels for glycolysis and tricarboxylic acid cycle to generate ATP, while using creatine-mediated ATP buffering system, a “molecular battery”, to store energy. This efficient “hybrid-engine” system enables tumor antigen-specific CD8 T cells to make maximal use of limited nutrients and mount their effector function in a metabolically challenging environment [35,39,59]. CD8 T cells rely heavily on the uptake of creatine from extracellular resources (Figure 1B). Therefore, manipulating creatine-mediated energy buffering system to reinvigorate tumor-fighting CD8 T cells could open up a new avenue in cancer immunotherapy. Although creatine can be taken from a creatine-rich diet as well as from dietary supplements (Figure 1C), creatine administration as a therapeutic intervention would generate the best antitumor benefits (Figure 1D).

In a mouse B16-OVA melanoma model, creatine supplementation either through i.p. injection or through oral administration effectively suppressed tumor growth, which was associated with a dramatic reduction in the number of PD-1^hi^CD62L^lo^ cells (an “exhaustion-prone” T cell phenotype) among the tumor-infiltrating CD8 T cells [44]. In another mouse syngeneic MC38 colon cancer model, animals receiving the creatine supplementation also had significantly reduced tumor growth compared to control animals [44], suggesting the creatine-induced tumor suppression effect is not tumor model specific and may be applicable to many different types of cancer. As the expression of CrT in the MC38 colon cancer cells is undetectable, the action of creatine supplementation is not on the tumor. In addition, creatine supplementation was not able to suppress B16-OVA tumor growth in immunodeficient NOD/SCID/IL-2Rγ^−/−^ (NSG) mice or in C57BL/6J WT mice with T cell depletion [44], suggesting that immune cells, especially T cells, may mediate the tumor suppression effect of creatine supplementation. Therefore, creatine supplementation has the translational potential as a new means of improving T cell antitumor activities for cancer immunotherapy.

If creatine provides a potent energy benefit for antitumor CD8 T cells, which is non-redundant to major immune checkpoint regulatory pathways, creatine supplementation may be a valuable component of combination immunotherapy to further enhance efficacy of current ICB treatments. Previous study has shown that the MC38 colon cancer model is responsive to PD-1/PD-L1 blockade therapy [60]. In this model, creatine supplementation in combination with anti-PD-1 therapy generated a more powerful antitumor effect compared to that of each monotherapy [44]. Of note, all the surviving mice from their primary tumors were protected against a second tumor challenge for another 6 months. This attractive antitumor effect was associated with significantly increased number of memory CD8^+^CD44^+^ T cells in the surviving mice [44]. Collectively, these encouraging findings point to a translational potential of creatine supplementation for combination cancer immunotherapy.

## 5. Creatine Supplementation for Cancer Therapy—Other Potential Benefits

In addition to its potential application for cancer immunotherapy, creatine supplementation has been indicated to augment the efficacy of the anticancer drug methylglyoxal (MG) [61]. Pal et al. reported that administration of creatine enhanced the antitumor effects of MG and ascorbic acid in sarcoma animal model in vivo and tumor burden was completely eradicated [62]. These results suggest that creatine supplementation can function as an adjunctive therapeutic intervention with other anticancer agents. Moreover, given the beneficial effects of creatine on muscle mass and physical function, emerging evidence has suggested that creatine supplementation may also have therapeutic potential for attenuating cancer-related weight loss and maintaining muscle function for cancer patients [63,64]. However, application in various cancer contexts has just began and further studies are needed to fully understand the impact of creatine supplementation on clinical outcomes in the cancer patient population at a risk of muscle wasting.

## 6. Creatine Supplementation for Cancer Therapy—Possible Influences on Cancer Cells

One of the hallmarks of cancer is the reprogramming of cellular metabolism [65]. In contrast to normal cells, cancer cells utilize cellular metabolites to support the high proliferation through distinct mechanisms in their local microenvironment [66]. Creatine-mediated ATP buffering system efficiently provides energy when cells demand high levels of ATP. Prior studies have shown that PCr can be used to transiently increase energy metabolism to promote cancer cell growth by buffering the ATP stores [67,68,69]. However, a number of studies suggest that creatine and its analogues can suppress tumor growth [1,61,62,70,71]. It has been initially demonstrated that creatine analogue cyclocreatine has antitumor properties in vitro [72]. A possible mechanism of antitumor effect is that phosphocyclocreatine generated from cyclocreatine by CK has a poor substrate activity in the CK reverse reaction and results in energy depletion in cancer cells [73]. However, cyclocreatine also suppressed tumor growth in colon adenocarcinoma without indications of energy depletion in cancer cells [70]. In line with this study, creatine has been shown to inhibit the growth and progression of mammary tumors, sarcoma and neuroblastoma tumors in both rats and mice models [71]. Creatine combined with MG, an anticancer drug, induced higher cytotoxicity and apoptosis in the human breast cancer MCF-7 cell line and chemically transformed the mouse C2C12 muscle cell line, compared to MG alone [62]. In contrast, no detrimental effects were observed in normal C2C12 muscle cells treated with MG plus creatine [62], suggesting that enhanced cytotoxic effects of MG plus creatine are specifically limited within cancer cells. Several lines of evidence have indicated that tumor creatine content is low in multiple types of cancer tissue [61,62,74,75]. Creatine content was restored to almost normal levels with the concomitant regression of tumor cells after creatine treatment [61,75]. Both creatine and cyclocreatine exhibit antitumor effects under certain tumor conditions, suggesting that creatine may employ additional mechanisms, which are independent of sustaining cellular energy charge, to mediate its antitumor effects.

A number of studies have linked cancer to creatine by investigating CK expression and their association with prognosis of cancer patients [76,77,78,79,80,81]. The expression of CKB was found to be upregulated in different types of cancer [76,78,79,80,81]. In addition, elevated expression of CKMT1 was correlated with poor prognosis in patients with breast cancer [76] or liver cancer [78]. Of note, the proliferation of cancer cells expressing high levels of CKB can be greatly impaired by cyclocreatine; however, the cancer cells with low levels of CK were resistant to the antitumor effect of cyclocreatine [72]. Overexpression of CKB in the cancer cells that expressed low levels of CK increased their sensitivity to cyclocreatine inhibition [72]. In the liver microenvironment, CKB released by metastatic colorectal cancer cells could use extracellular ATP to phosphorylate hepatocyte-secreted creatine to produce PCr. Metastatic cells then use PCr to regenerate ATP to support their survival in the liver [82]. Although the source for extracellular ATP is not clear, these data suggest that a crosstalk between normal cells and cancer cells is necessary to support PCr-mediated cancer cell survival under certain conditions. Supplementing PCr to colorectal cancer cells with ATP depletion rescued their ATP levels [82]. The mechanism of PCr atypically imported by CrT on cancer cells remains to be determined.

Abnormal gene expression of ecotropic virus integration site-1 (EVI1), an oncogenic transcription factor, was observed in some patients with acute myeloid leukemia (AML) and was correlated with poor survival of patients [83]. The EVI1-positive AML subtype has a poor response to current treatment regimens [84]. By using a screen of pooled shRNAs, Fenouille and colleagues have demonstrated that CKMT1 contributes to survival of EVI1-expressing cells in EVI1-positive AML patients [68]. EVI1 has been suggested to promote CKMT1 expression by suppressing the myeloid differentiation regulator RUNX1. Inhibition of creatine metabolism by either CKMT1 depletion or using creatine analogue cyclocreatine specifically enhanced the cell cycle arrest and apoptotic cell death of EVI1-positive cancer cells, and increased animal survival in AML mouse syngeneic and xenograft models [68]. The blockade of CKMT1 impaired proliferative capacity, mitochondrial function, and ATP production in cancer cells, which could be rescued by exogenous PCr in vitro and in vivo [68]. Thus, these findings point to the therapeutic potential of targeting CKMT1 pathway for treating EVI1-positive AML.

Kurmi and colleagues have reported that oncogenic HER2 signaling induces CKMT1 phosphorylation via ABL tyrosine kinase in HER2 positive breast cancer cells [67]. CKMT1 phosphorylation enhanced the stabilization of CKMT1, promoting the PCr energy shuttle and breast cancer cell proliferation [67]. Blockade of the PCr-mediated metabolism by using either CKMT1 shRNA or the creatine analogue cyclocreatine decreased proliferation of HER2 positive cell lines in vitro and *in vivo*. The impaired proliferation could be rescued by PCr supplementation [67]. Although the HER2-directed monoclonal antibody trastuzumab offers significant clinical benefit selectively in HER2 positive breast cancer patients, intrinsic and acquired resistance to this therapy leads to no response for many patients [85,86,87,88,89]. Cyclocreatine combined with the HER2 kinase inhibitor lapatinib efficiently suppressed tumor growth in a HER2 positive trastuzumab-resistant patient-derived xenograft model [67]. Collectively, cyclocreatine can inhibit trastuzumab-resistant HER2 positive breast cancer cells via disrupting the PCr energy shuttle and improve the efficacy of existing breast cancer treatments.

Recently, Maguire et al. investigated the crosstalk between adipocytes and neoplastic cells in the tumor microenvironment and identified the upregulation of *Gatm* and the fatty acyl-CoA synthetase gene *Acsbg1* in adipocytes and in breast cancer cells, respectively [90]. Genetic inhibition of either *Gatm* in adipocytes or *CrT* in breast cancer cells attenuated tumor growth in obesity [90], suggesting that adipocyte-derived creatine is required for obesity-driven tumor progression. High *Acsbg1* expression in cancer cells enhanced ATP production through oxidative phosphorylation and uptake of adipocyte-derived creatine through CrT, which supported the production of PCr and drove tumor cell proliferation [90]. Although these findings revealed a protumoral role of creatine in regulating obesity-accelerated breast cancer cell proliferation, creatine supplementation did not promote tumor growth in lean or obese animals [90]. Thus, only adipocyte-derived creatine, not systemic creatine, is essential for tumor cell growth in this specific breast cancer model. Meanwhile, another group reported that creatine supplementation or GATM-mediated *de novo* synthesis of creatine promoted colorectal and breast cancer metastasis in orthotopic mouse models by increasing Snail and Slug expression through monopolar spindle 1 (MPS1)-activated Smad2 and Smad3 phosphorylation [91]. Moreover, GATM knockdown or MPS1 inhibition attenuated cancer metastasis and lower survival in mice. Notably, creatine supplementation suppressed primary tumor growth in mouse colon cancer (CT26) and mouse breast cancer (4T1) models [91]. Several notes of caution should be made in interpreting these results of cancer metastasis: (1) some studies were performed using severely immunocompromised mice (i.e., the NSG mice) and therefore failed to take into account the creatine regulation of antitumor immunity effect [44]; (2) creatine supplementation doses used in some studies were exceedingly high, way above the recommended safe dose range for humans [10,92]; and (3) long-term creatine supplementation has been indicated to induce species-specific liver inflammation in mice, which could contribute to promoting cancer metastasis in liver observed in these studies [93,94,95].

## 7. Conclusions

Recent studies identified creatine as an important metabolic regulator conserving bioenergy to power CD8 T cell antitumor immunity and suggested a potential application of creatine supplementation for cancer immunotherapy. The safety of long-term creatine supplementation in both healthy individuals and patients has been well documented, which provides a clear and expedient path forward for utilizing creatine supplementation to treat cancer [13,64,96,97,98,99]. Additionally, creatine supplementation can augment muscle and enhance strength, which may also benefit cancer patients suffering from cachexia at their late stages [63,64,100,101]. Both oral and intravenous administration routes can be effective in animal tumor models [44]. However, bioavailability of creatine through oral administration in humans is low because creatine is rapidly converted into creatinine in the high acidic environment of the stomach [102,103]. Thus, for the best cancer therapy benefits, optimal administration routes and dosing strategies for creatine clinical intervention still need to be further investigated.

Creatine supplementation synergizes with the anti-PD-1 therapy to yield superior antitumor efficacy [44], because creatine may activate tumor infiltrating CD8 T cells via an energy-buffering mechanism that is non-redundant to the mechanisms utilized by ICB therapies. Therefore, creatine supplementation has a potential to become an economical and effective treatment for enhancing ICB therapies. In addition, many other cancer therapies, including new T cell immunotherapies, traditional chemotherapy, targeted therapy, and radiation therapy, may also have improved therapeutic efficacy when combined with creatine supplementation treatment [31,32,33,34,67,104,105,106]. For the full translation to clinical applications, a speedy clinical development of creatine supplementation for combination therapies treating different types of cancer is urgently needed in the future.

Creatine-mediated energy buffering system is efficient and supports CD8 T cell antitumor activities in a metabolically challenging microenvironment via ATP/AMPK-mediated regulation of TCR signaling pathways [35,39,44,59]. The hyporesponsiveness of other immune cells in *CrT*-KO mice bearing tumors has been observed, indicating that the immune regulatory function of this energy system may go beyond modulating CD8 T cells in a tumor microenvironment. Creatine has been indicated to have anti-inflammatory properties in acute and chronic animal models of inflammation [107]. Creatine uptake can reprogram macrophage M1/M2 polarization by regulating IFN-γ and IL-4 cytokine responses partly in an ATP-dependent manner under infection conditions [108]. Whether and how other immune cells, such as regulatory T cells and tumor-associated macrophages, in the tumor microenvironment mediate antitumor effects of creatine will be interesting topics and certainly merit investigation. Additionally, creatine and creatine analogues can inhibit tumor cell survival likely through additional mechanisms that are independent of energy metabolism disruption. The regulatory mechanisms of creatine and creatine analogues in cancer cells warrant further investigation, especially in light of the recent studies on creatine promotion of cancer metastasis.

## Data Availability

Not applicable.
